# Supplementary material for: Next-Generation Protein Sequencing and individual ion mass spectrometry enable complementary analysis of interleukin-6
Source: bioRxiv. 2025 Feb 8:2025.02.07.637157. Preprint. [Version 1] doi: 10.1101/2025.02.07.637157 (PMC11839055; doi:10.1101/2025.02.07.637157)
Supplement: Supplement 1 [file NIHPP2025.02.07.637157v1-supplement-1.pdf]

bioRxiv preprint doi: https://doi.org/10.1101/2025.02.07.637157; this version posted February 8, 2025. The copyright holder for this preprint (which was not certified by peer review) is the author/funder, who has granted bioRxiv a license to display the preprint in perpetuity. It is made available under aCC-BY 4.0 International license.

Supplementary Information

Next-Generation Protein Sequencing and individual ion mass spectrometry enable complementary analysis of interleukin-6

Kenneth A. Skinner[1]; Troy D. Fisher[2]; Andrew Lee[3]; Taojunfeng Su[3]; Eleonora Forte[2,4]; Aniel Sanchez[2]; Michael A. Caldwell[2,5]; Neil L. Kelleher[2,3,6#]

[1]Quantum-Si, Incorporated, Branford, Connecticut 06405
[2]Proteomics Center of Excellence, Northwestern University, Evanston, Illinois 60208
[3]Departments of Molecular Biosciences, Chemistry and Chemical and Biological Engineering, Northwestern University, Evanston, IL, USA
[4]Department of Surgery, Feinberg School of Medicine, Comprehensive Transplant Center, Northwestern University, Chicago, Illinois 60611, United States
[5]Department of Medicine, Division of Hematology Oncology, Feinberg School of Medicine, Northwestern University, Chicago, IL, USA
[6]Department of Biochemistry and Molecular Genetics, Feinberg School of Medicine, Northwestern University, Chicago, IL, USA

#Corresponding author email: n-kelleher@northwestern.edu

bioRxiv preprint doi: https://doi.org/10.1101/2025.02.07.637157; this version posted February 8, 2025. The copyright holder for this preprint (which was not certified by peer review) is the author/funder, who has granted bioRxiv a license to display the preprint in perpetuity. It is made available under aCC-BY 4.0 International license.

Rank	Inferred Protein	Score	Likelihood	Mass (kDa)	Length	Digested Peptides	Inferred Peptides
1	sp|P05231|IL6_HUMAN	9.222594	99.99%	24	212	5	5
2	sp|Q6NXT1|ANR54_HUMAN	0.257477	22.7%	32	300	1	1

Supplementary Figure 1. Protein inference v2.5.2 aligns observed kinetic signatures from sequencing data of rhIL-6 to a database of predicted kinetic signatures. Protein Score is an estimate of the likelihood of protein identity. While ankyrin repeat domain-containing protein 54 (UniProt ID: Q6NXT1) contains peptide QIIHMLREYLERLGQHEQRERLDDLCTRLQMTSTK, which is similar in sequence to QIRYILDGISALRK in rhIL-6, Platinum unambiguously identifies rhIL-6.

Supplementary Table 1. Intact Proteoforms of rhIL-6 detected with I^2MS. 13 proteoforms of rhIL-6 were detected by I^2MS and abundant species were targeted for tandem-MS with I^2MS^2. Based on the intact masses, putative glycan compositions were proposed and supported with I^2MS^2 data when available.

PFR	Theoretical Monoisotopic Mass (Da)	Observed Monoisotopic Mass (Da)	Mass Error (ppm)	Half Cystine Sites	Putative O-Glycan Composition	Glycan Chemical Formula (unbound)	Monoisotopic Glycan Mass (Da)	I^2MS^2?
1	20795.58	20795.55	-1.55	C72, C78, C101, C111	N/A	N/A	N/A	Yes
2	20998.66	20998.64	-1.03	C72, C78, C101, C111	HexNAc(1)	C8H13NO5	+221.08994	No
3	21160.71	21160.69	-1.16	C72, C78, C101, C111	HexNAc(1) Hex(1)	C14H23NO10	+383.14276	No
4	21289.76	21289.72	-1.74	C72, C78, C101, C111	HexNAc(1) NeuAc(1)	C19H30N2O13	+512.18535	Yes
5	21452.17	21451.79	-17.71	C72, C78, C101, C111	HexNAc(1) Hex(1) NeuAc(1)	C25H40N2O18	+674.23818	Yes
6	21646.87	21646.84	-1.52	C72, C78, C101, C111	HexNAc(1) Hex(4)	C32H53NO25	+869.30123	No
7	21654.89	21654.86	-1.35	C72, C78, C101, C111	HexNAc(2) Hex(1) NeuAc(1)	C33H53N3O23	+877.31755	No
8	21742.91	21742.90	-0.25	C72, C78, C101, C111	HexNAc(1) Hex(1) NeuAc(2)	C36H57N3O26	+965.33359	Yes
9	21785.95	21785.89	-2.64	C72, C78, C101, C111	HexNAc(4) Hex(1)	C38H62N4O25	+992.38088	Yes
10	21858.99	21858.91	-3.62	C72, C78, C101, C111	HexNAc(3) dHex(2) Hex(1)	C42H69N3O28	+1081.41732	No
11	21905.98	21905.95	-1.30	C72, C78, C101, C111	HexNAc(1) dHex(2) Hex(2) NeuAc(1)	C43H70N2O31	+1128.40682	Yes
12	21947.01	21946.97	-1.60	C72, C78, C101, C111	HexNAc(2) dHex(2) Hex(1) NeuAc(1)	C45H73N3O31	+1169.43337	Yes
13	22109.06	22109.03	-1.26	C72, C78, C101, C111	HexNAc(2) dHex(2) Hex(2) NeuAc(1)	C51H83N3O36	+1331.48619	Yes

bioRxiv preprint doi: https://doi.org/10.1101/2025.02.07.637157; this version posted February 8, 2025. The copyright holder for this preprint (which was not certified by peer review) is the author/funder, who has granted bioRxiv a license to display the preprint in perpetuity. It is made available under aCC-BY 4.0 International license.

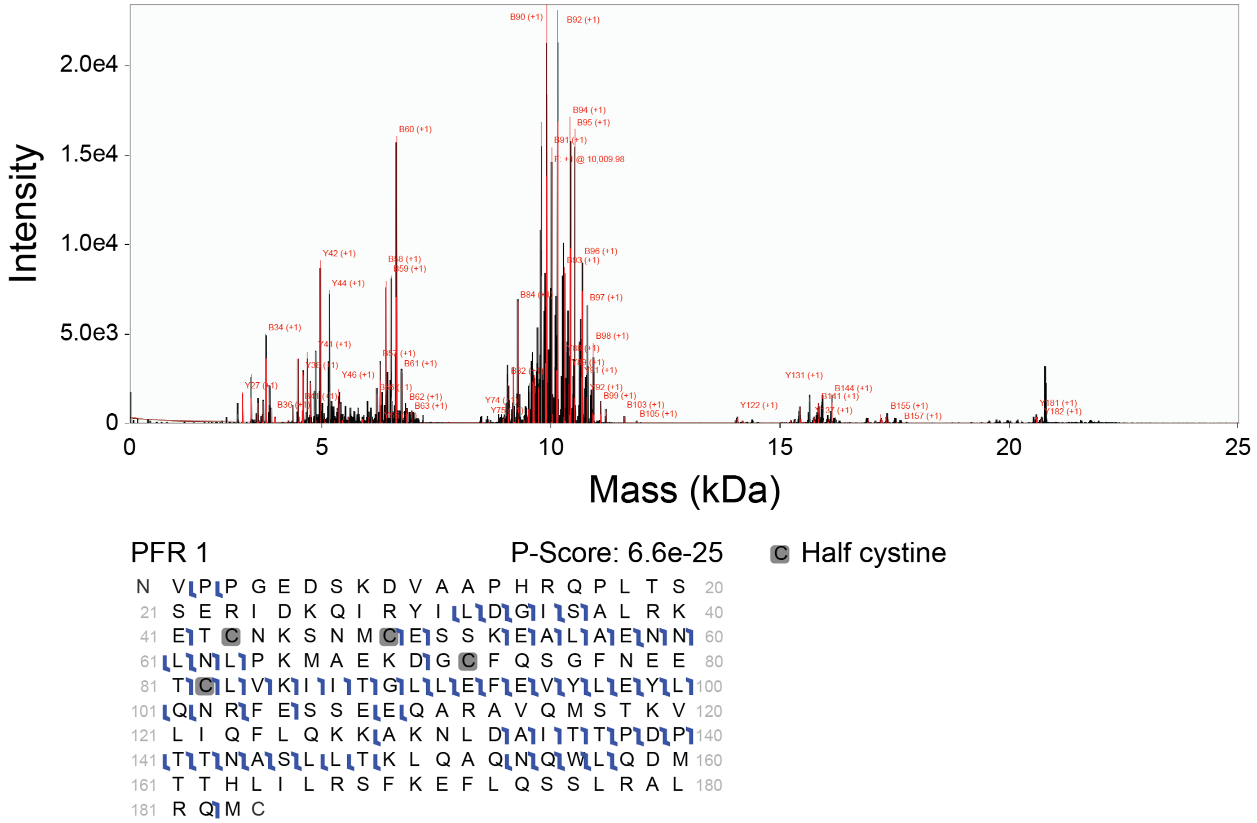

Supplementary Figure 2. Fragmentation spectrum and graphical fragment map for rhIL-6 proteoform 1.

bioRxiv preprint doi: https://doi.org/10.1101/2025.02.07.637157; this version posted February 8, 2025. The copyright holder for this preprint (which was not certified by peer review) is the author/funder, who has granted bioRxiv a license to display the preprint in perpetuity. It is made available under aCC-BY 4.0 International license.

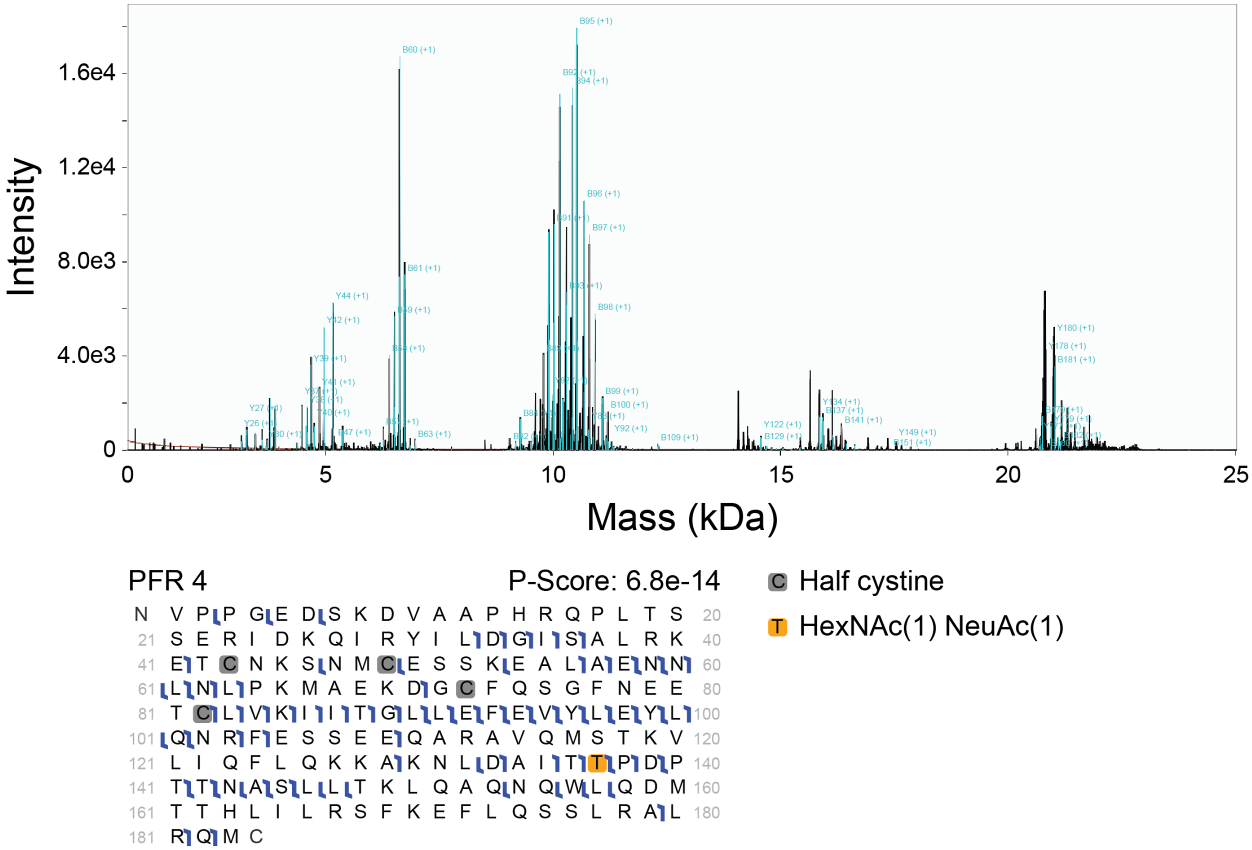

Supplementary Figure 3. Fragmentation spectrum and graphical fragment map for rhIL-6 proteoform 4.

bioRxiv preprint doi: https://doi.org/10.1101/2025.02.07.637157; this version posted February 8, 2025. The copyright holder for this preprint (which was not certified by peer review) is the author/funder, who has granted bioRxiv a license to display the preprint in perpetuity. It is made available under aCC-BY 4.0 International license.

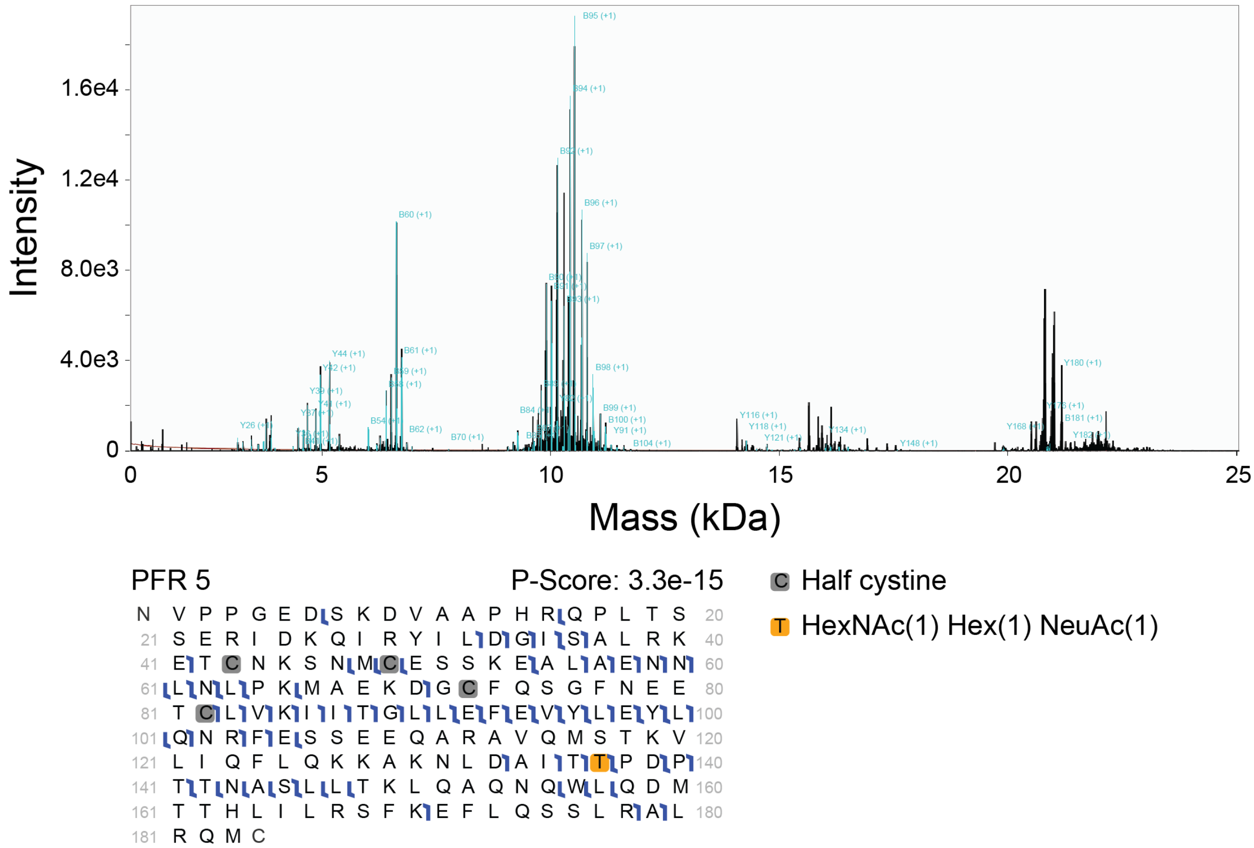

Supplementary Figure 4. Fragmentation spectrum and graphical fragment map for rhIL-6 proteoform 5.

bioRxiv preprint doi: https://doi.org/10.1101/2025.02.07.637157; this version posted February 8, 2025. The copyright holder for this preprint (which was not certified by peer review) is the author/funder, who has granted bioRxiv a license to display the preprint in perpetuity. It is made available under aCC-BY 4.0 International license.

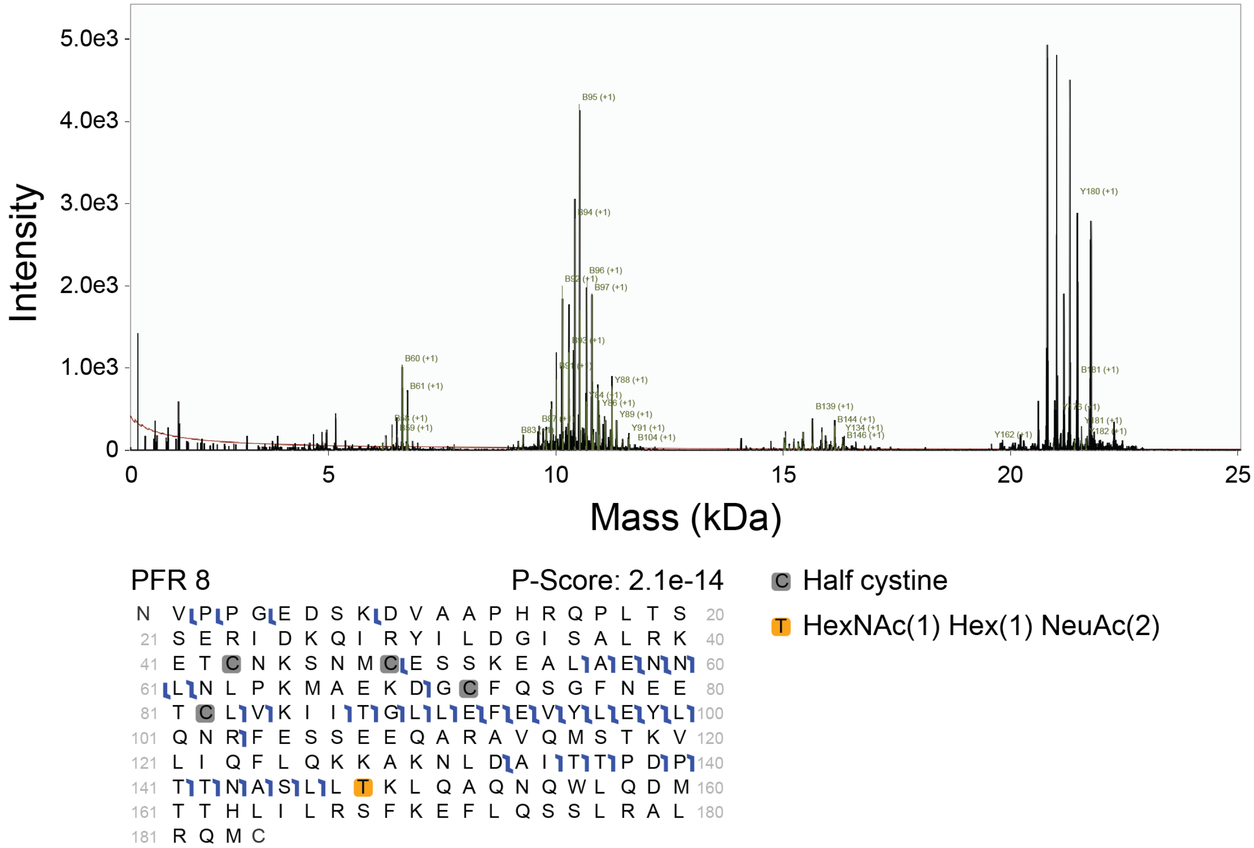

Supplementary Figure 5. Fragmentation spectrum and graphical fragment map for rhIL-6 proteoform 8.

bioRxiv preprint doi: https://doi.org/10.1101/2025.02.07.637157; this version posted February 8, 2025. The copyright holder for this preprint (which was not certified by peer review) is the author/funder, who has granted bioRxiv a license to display the preprint in perpetuity. It is made available under aCC-BY 4.0 International license.

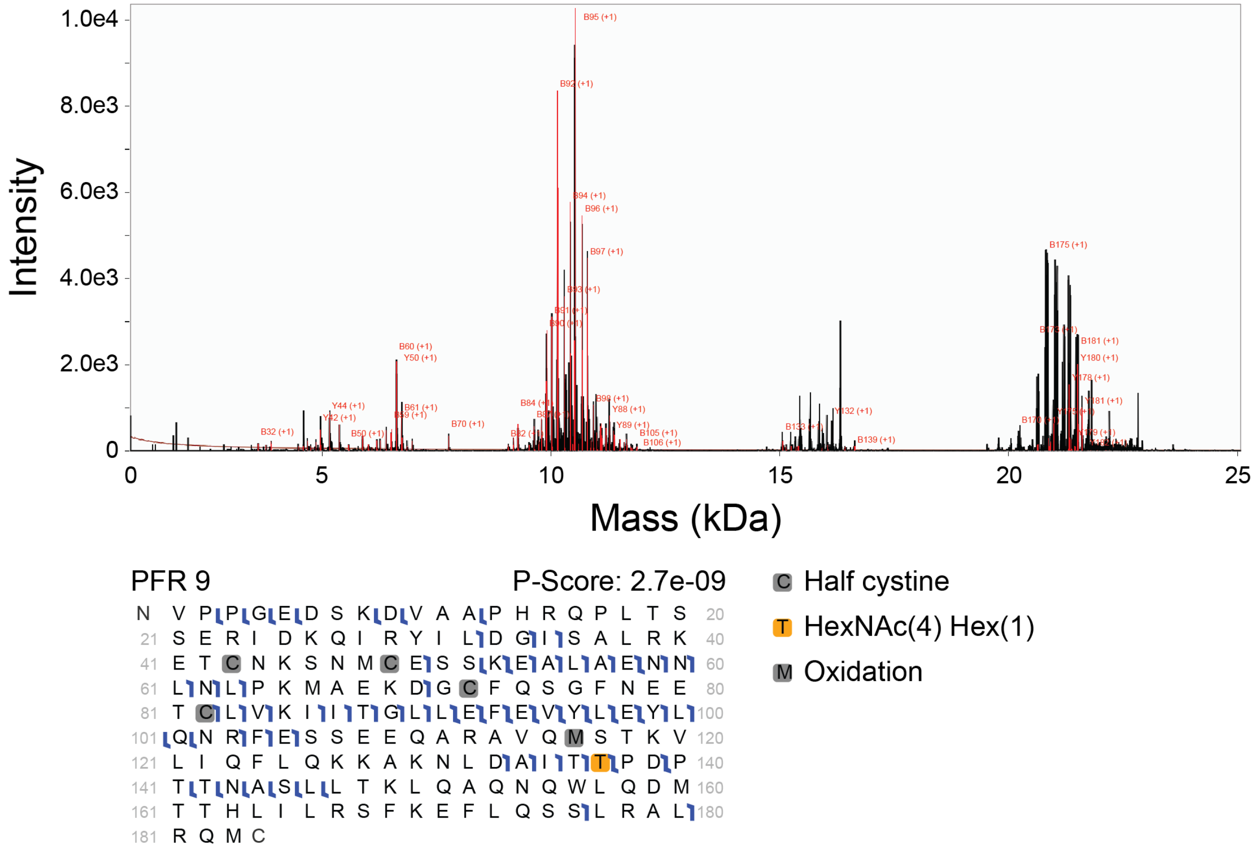

Supplementary Figure 6. Fragmentation spectrum and graphical fragment map for rhIL-6 proteoform 9.

bioRxiv preprint doi: https://doi.org/10.1101/2025.02.07.637157; this version posted February 8, 2025. The copyright holder for this preprint (which was not certified by peer review) is the author/funder, who has granted bioRxiv a license to display the preprint in perpetuity. It is made available under aCC-BY 4.0 International license.

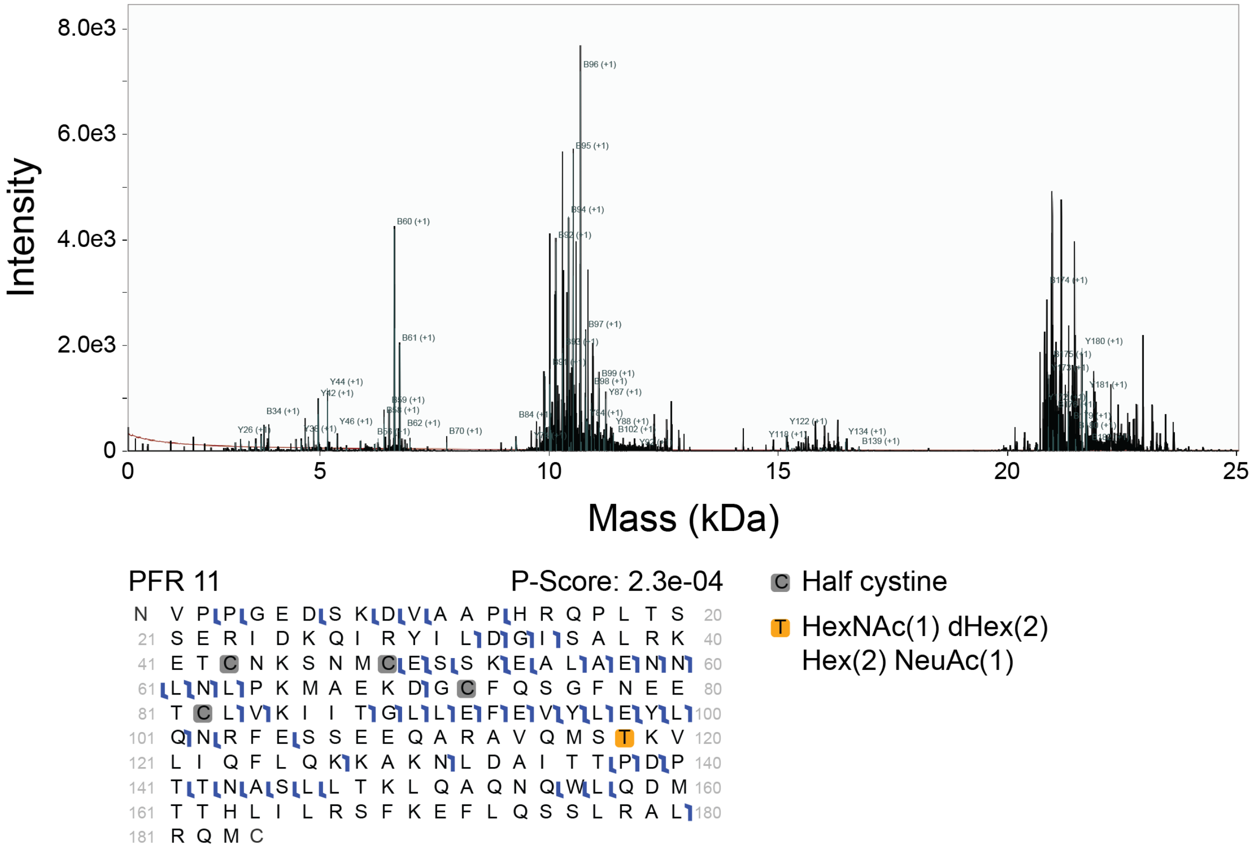

Supplementary Figure 7. Fragmentation spectrum and graphical fragment map for rhIL-6 proteoform 11.

bioRxiv preprint doi: https://doi.org/10.1101/2025.02.07.637157; this version posted February 8, 2025. The copyright holder for this preprint (which was not certified by peer review) is the author/funder, who has granted bioRxiv a license to display the preprint in perpetuity. It is made available under aCC-BY 4.0 International license.

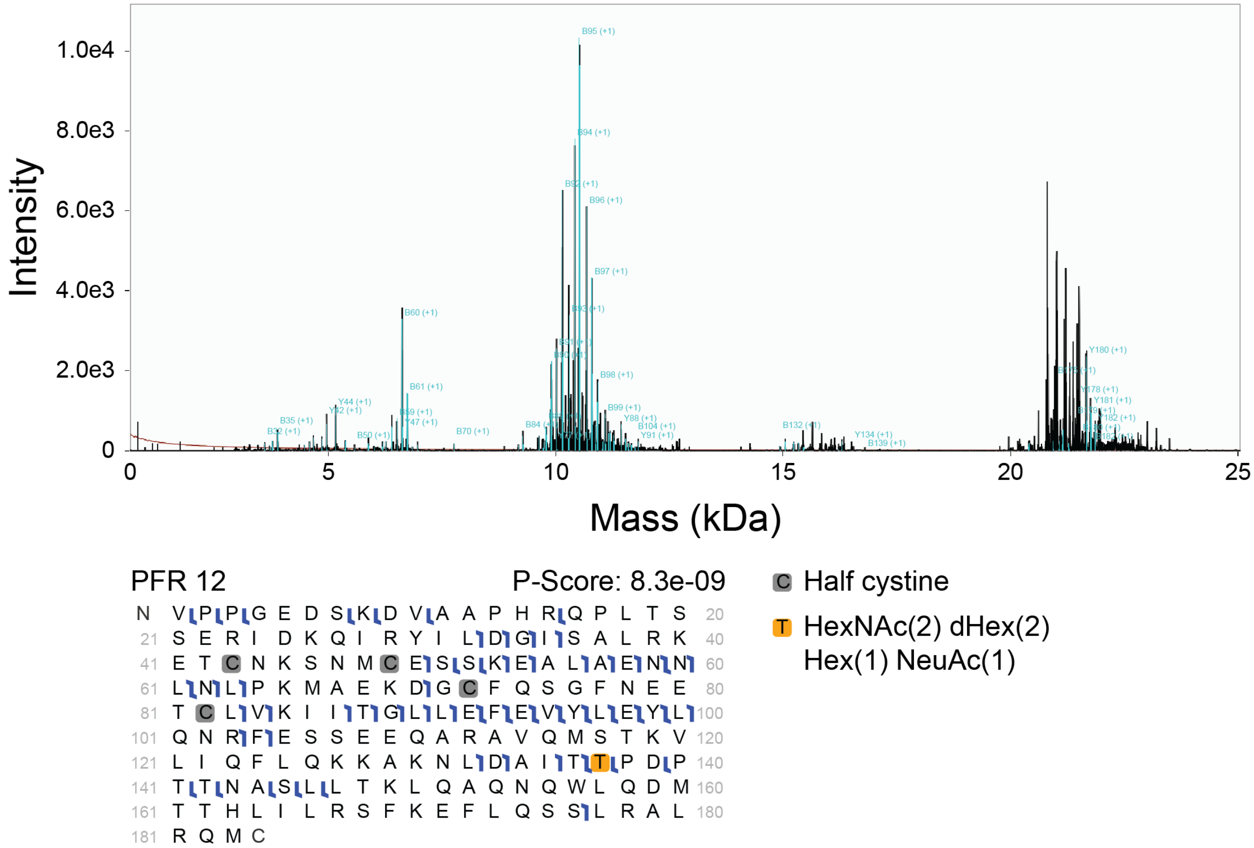

Supplementary Figure 8. Fragmentation spectrum and graphical fragment map for rhIL-6 proteoform 12.

bioRxiv preprint doi: https://doi.org/10.1101/2025.02.07.637157; this version posted February 8, 2025. The copyright holder for this preprint (which was not certified by peer review) is the author/funder, who has granted bioRxiv a license to display the preprint in perpetuity. It is made available under aCC-BY 4.0 International license.

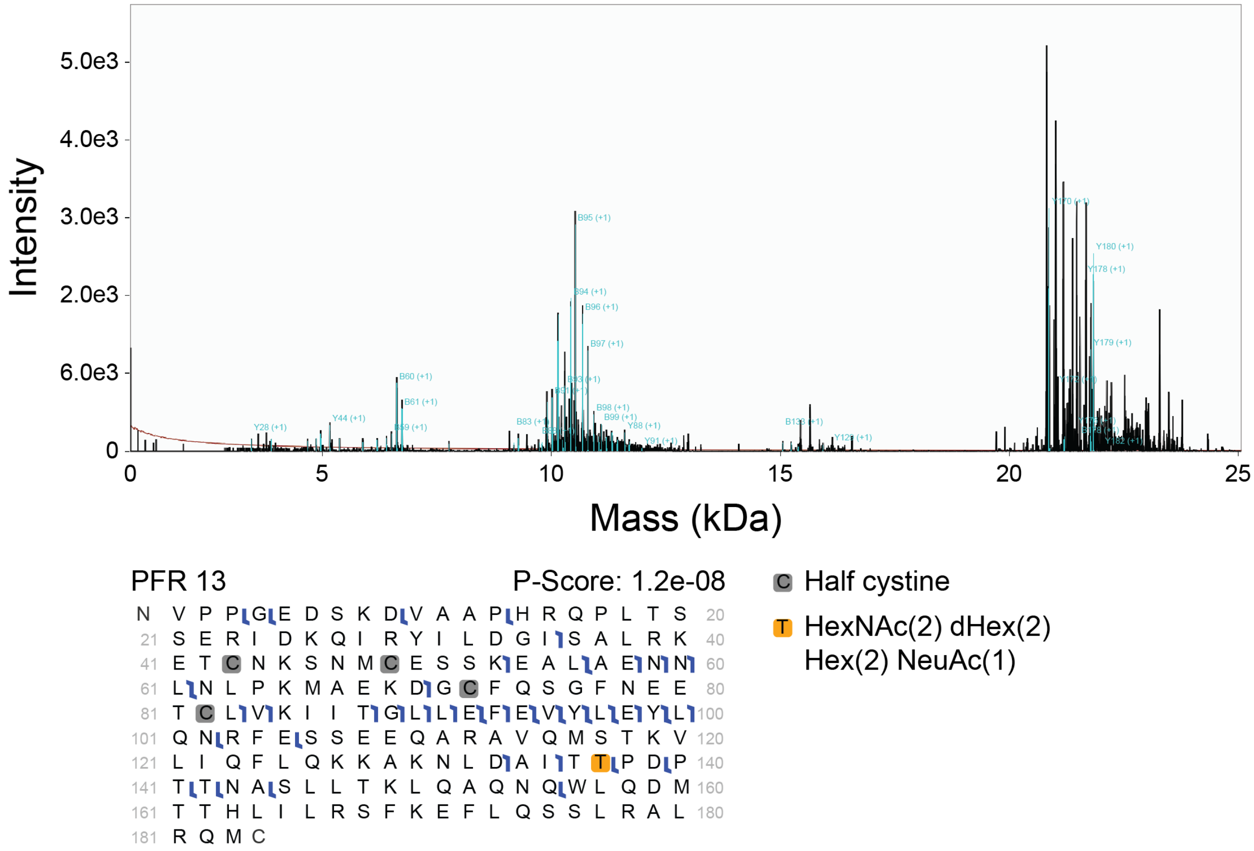

Supplementary Figure 9. Fragmentation spectrum and graphical fragment map for rhIL-6 proteoform 13.
